# Treatment with high-dose recombinant human hyaluronidase-facilitated subcutaneous immune globulins in patients with juvenile dermatomyositis who are intolerant to intravenous immune globulins: a report of 5 cases

**DOI:** 10.1186/s12969-016-0112-6

**Published:** 2016-09-13

**Authors:** Fabian Speth, Johannes-Peter Haas, Claas H. Hinze

**Affiliations:** 1 Deutsches Zentrum für Kinder- und Jugendrheumatologie, Garmisch-Partenkirchen, Germany; 2 Klinik für Pädiatrische Rheumatologie und Immunologie, Universitätsklinikum Münster, Münster, Germany

**Keywords:** Juvenile dermatomyositis, Intravenous immune globulins, Subcutaneous immune globulins, Recombinant human hyaluronidase

## Abstract

**Background:**

High-dose intravenous immune globulins (IVIg) are frequently used in refractory juvenile dermatomyositis (JDM) but are often poorly tolerated. High-dose recombinant human hyaluronidase-facilitated subcutaneous immune globulins (fSCIg) allow the administration of much higher doses of immune globulins than conventional subcutaneous immune globulin therapy and may be an alternative to IVIg. The safety and efficacy of fSCIg therapy in JDM is unknown.

**Case Presentation:**

In this retrospective case series, five patients with steroid-refractory severe JDM were treated with high-dose fSCIg due to IVIg adverse effects (severe headaches, nausea, vomiting, difficult venous access). Peak serum IgG levels, muscle enzymes, the childhood myositis assessment scale and adverse effects were retrieved for at least 6 months following intiation of fSCIg. Data were analyzed by descriptive statistics.

Patients initially received fSCIg 1 g/kg every 14 days, resulting in median IgG peak levels of 1901 mg/dl (1606–2719 mg/dl), compared to median IgG peak and trough levels while previously receiving IVIg of 2741 mg/dl (2429–2849 mg/dl) and 1351 mg/dl (1156–1710 mg/dl). Additional antirheumatic therapies consisted of low-dose glucocorticoid therapy, methotrexate, mycophenolate mofetil and/or rituximab. Two patients maintained clinically inactive disease and three patients had only a partial treatment response. In the three patients with partial treatment response, fSCIg 1 g/kg was then given on days 1 and 6 of every 28-day cycle resulting in IgG peak levels of between 2300–2846 mg/dl (previously 1606–1901 mg/dl on the biweekly regimen), resulting in clinically inactive disease in two of the three patients. There were no relevant adverse effects that limited continuation of fSCIg treatment.

**Conclusions:**

High-dose fSCIg is well-tolerated in patients with JDM and high peak serum IgG levels can be achieved which may be important for treatment success. High-dose fSCIg may therefore be an alternative to high-dose IVIg and deserves further study.

**Trial registration:**

This is a case series and data were retrospectively registered.

## Background

Juvenile dermatomyositis (JDM) is a severe inflammatory myopathy characterized by vasculopathy affecting skin, muscle and sometimes internal organs [1]. While mortality is low with contemporary treatment, morbidities, such as dystrophic calcification, contractures and muscle weakness still cause a substantial long-term disease burden [2, 3]. However, little high-quality data from clinical trials exists to guide treatment decisions and treatment is mostly guided by expert opinion and consensus treatment protocols [1, 4–6]. There is evidence that the routine treatment of moderately-severe JDM should include high-dose steroids and methotrexate [7]. Additional steroid-sparing immunomodulatory therapies are often employed, especially including intravenous immune globulins (IVIg), hydroxychloroquine, cyclosporine, azathioprine, mycophenolate mofetil and rituximab. Even though randomized clinical trials for IVIg in JDM are lacking, it appears to be highly effective in severe or refractory JDM [8, 9]. Typically, the administration of up to 2 g per kg and month of IVIg are required to achieve treatment success, since anti-inflammatory efficacy may depend on peak serum immune globulin (Ig)G levels [10, 11]. High-dose IVIg treatment causes adverse effects in 5-10 % of patients [11], including severe headaches, nausea, vomiting, aseptic meningitis, thrombosis and venous access may be difficult over time whereas subcutaneous Ig (SCIg) treatment is usually well-tolerated [12]. However, since the extracellular matrix limits subcutaneous bulk fluid flow, the maximal volume infusible by standard SCIg in one site is 30 ml, effectively limiting the amount of Ig that can be applied in a single treatment and, thus, its utility as an anti-inflammatory therapy. By means of facilitating SCIg with recombinant human hyaluronidase (rHUPH20) (fSCIg), a 20-fold higher volume of up to 600 ml fSCIg (equaling 60 g of Ig) can be administered at one site [13]. Cleaving hyaluronic acid increases permeability of the subcutaneous tissue markedly. Since rHUPH20 is not available systemically, short-acting and non-immunogenic, and since tissue hyaluronic acid is rapidly resynthesized, there are typically no short-term or long-term adverse effects. Regarding the pharmacokinetics of fSCIg, the median time to reach peak serum IgG levels is five days [13]. fSCIg is currently approved for the use in patients with primary immunodeficiency disorders older than 18 years of age but has been tested in children older than two years of age. The safety and efficacy of high-dose fSCIg therapy in JDM is unknown.

## Case Presentation

### Patients

Five patients with moderately severe or severe and refractory definitive JDM who had initially received IVIg were treated with fSCIg for at least six months were identified at the German Center for Pediatric and Adolescent Rheumatology in Garmisch-Partenkirchen between January 2012 and December 2015 [5]. Overall, 42 patients with JDM were treated at the center in that time frame, of whom 26 had received IVIg (62 %). Informed consent was obtained from all patients and guardians. Patient demographics, myositis-specific autoantibodies, clinical phenotype, disease severity [5], concurrent antirheumatic therapy and reasons for initiation of/switching to fSCIg treatment are shown in Table 1.

**Table 1:** Patient demographics, current antirheumatic therapy, reasons for initiation of subcutaneous immune globulins, and disease course following fSCIg therapy

Patient	Sex, age, age at disease onset, body weight	Clinical phenotype and disease severity^a^, age at disease onset, MSA status	Disease activity at time of fSCIg initiation	Previous or concurrent antirheumatic treatments	Reason for initiation of fSCIg instead of IVIg, CMAS at time of fSCIg initiation	Disease course following initiation of fSCIg
1	Male, 11 years, 9 years at disease onset, 35 kg	Classic, severe JDM with prominent skin involvement, joint contractures, Anti-Mi-2 positive	Clinically inactive (however, recent recurrence after attempted IVIg discontiuation)	IVIg (60 g/month), fSCIg (70 g/month), PDN 0.125 mg/kg/day, MTX 15 mg/m^2^/week, HCQ	IVIg adverse effects (headaches, nausea and vomiting), needle phobia CMAS 51	Within 3 months mild deterioration of CMAS (49 instead of 51); resolution after switching to fSCIg 5 days apart
2	Female, 10 years, 8 years at disease onset, 35 kg	Classic, severe JDM with prominent vaculopathy, dysphagia, cutaneous ulcerations, myocarditis, joint contractures, calcinosis, Anti-TIF-1gamma positive	Residual disease activity, vasculopathy	IVIg (70 g/month), fSCIg (70 g/month), PDN 0.21 mg/kg/day, MMF 1200 mg/m^2^/d, MTX 15 mg/m^2^/week, RTX (status post 375 mg/m^2^ ×4), CYC (status post 6 × 750 mg/m^2^)	IVIg adverse effects (headaches), difficult peripheral venous access with port-a-cath CMAS not interpretable due to severity of contractures	Stable mild residual disease activity even after switching to fSCIg 5 days apart
3	Female, 7 years, 5 years at disease onset, 20 kg	Classic, severe JDM with prominent vasculopathy, cutaneous ulcerations, Anti-SRP and anti-MI-2 positive	Clinically inactive (recent recurrence)	IVIg (40 g/month), fSCIg (40 g/month), PDN 0.125 mg/kg/day, MMF 1200 mg/m^2^/d, MTX 15 mg/m^2^/week RTX (status post 375 mg/m^2^ × 4 [first cycle], 750 mg/m^2^ × 1 [second cycle])	IVIg adverse effects (headaches, nausea and vomiting), needle phobia CMAS 52	Maintenance of clinically inactive disease; CMAS 52; fSCIg continued biweekly
4	Male, 12 years, 8 years at disease onset, 35 kg	Classic, moderately severe JDM with nodular dystrophic calcification, anti-NXP-2 positive	Clinically inactive (however, progressive calcinosis)	IVIg (35 g/month), fSCIg (60 g/month) PDN 0.07 mg/kg/day, MMF 1200 mg/m^2^/d, Colchicin	IVIg adverse effects (headaches, nausea and vomiting), needle phobia, residual disease activity with insufficient IVIg dose CMAS 52	Maintenance of clinically inactive disease; CMAS 52; calcinosis decreasing; fSCIg continued biweekly
5	Female, 8 years, 5 years at disease onset, 20 kg	Classic, severe JDM with rhadomyolysis, failure to thrive and nephrolithiasis, MSA negative	Active skin and muscle disease (IVIg was discontinued 6 months earlier)	IVIG (40 g/month), fSCIg (50 g/month) PDN 0.19 mg/kg/day, MMF 600 mg/m^2^/d (diarrhea with higher doses), MTX 15 mg/m^2^/week	IVIg adverse effects (headaches, nausea and vomiting), needle phobia CMAS 46	Improvement but residual, mild proximal muscle weakness; normal muscle strength (inactive disease) after switching to fSCIg 5 days apart; CMAS 50; further PDN reduction

*Abbreviations: CYC* cyclophosphamide, *IVIg* intravenous immune globulins, *fSCIg* subcutaneous immune globulins facilitated by recombinant human hyaluronidase, *MMF* mycophenolate mofetil, *MSA* myositis-specific antibodies, *MTX* methotrexate, *PDN* prednisolone, *RTX* rituximab

^a^Definition of disease severity according to Huber et al. [5]

### Administration of subcutaneous immune globulins

Patients 1–4 started fSCIg treatment 4–6 weeks after the last IVIg dose, and patient 5, who had not recently received IVIg, started fSCIg after a disease flare. All patients received high-dose fSCIg with recombinant human hyaluronidase (HyQvia, Baxalta, Unterschleißheim, Germany) between 1.7 to 2 g/kg per month (maximally 70 g per month) divided into two doses. Patients each received two inpatient training fSCIg treatments (first 0.3 g/kg, then 1 g/kg). Following hospital discharge, patients and parents received one or two fSCIg treatments at home guided by a nurse practitioner especially trained in the application of fSCIg. Eutectic mixture of local anesthetics was applied prior to the subcutaneous infusions and fSCIg was administered according to the manufacturer’s instruction with maximal infusion rates of 160 ml/h (body weight <40 kg). Each individual fSCIg administration took on average three to four hours. For three patients, the regimen was later switched to two monthly fSCIg doses five days apart, i.e., the same monthly total dose was given divided into two doses at days 1 and 6 of each 28-day cycle.

### Data collection and analysis

As part of the clinical routine at the center multiple clinical and laboratory parameters are collected prospectively for all patients with JDM. For this retrospective analysis, the following clinical and laboratory parameters were retrieved for analysis: serum IgG levels, muscle enzyme levels, childhood myositis assessment scale (CMAS) score, physician global assessment of disease activity, and potential treatment adverse effects. Data were analyzed using descriptive statistics.

### Serum IgG levels

**Fig. 1:**
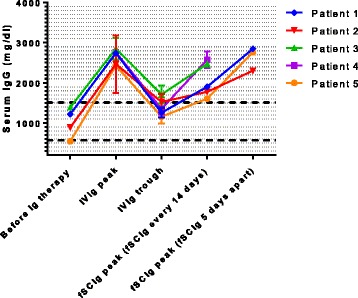
IgG levels before initiation of Ig therapy, peak and trough serum IgG levels (during high-dose IVIg therapy) and peak levels during SCIg therapy. Values represent mean values and error bars standard deviation (if multiple measurements are available). The *dashed lines* represent the upper and lower limit of the normal range

For the various time points of measuring serum IgG levels (before Ig therapy, peak/trough during IVIg, and peak during SCIg) up to nine different data point were available and mean IgG levels were calculated for each patient and time point. Overall, fSCIg treatment every 14 days resulted in median IgG peak levels, measured five days after administration, of 1901 mg/dl (range 1606–2719 mg/dl), compared to median IgG peak (one day after dose) and trough levels (28 days after dose) while previously receiving IVIg 2 g per kg and month of 2741 mg/dl (range 2429–2849 mg/dl) and 1351 mg/dl (1156–1710 mg/dl), respectively. In order to achieve higher peak serum levels and improved immunomodulatory efficacy, for three patients the fSCIg administration was switched to two monthly doses five days apart (i.e., days 1 and 6 of each 28-day cycle). Following fSCIg 1 g/kg on days 1 and 6, IgG serum levels on day 11 showed much increased IgG serum levels of 2846 mg/dl (up from 1901 mg/dl), 2300 mg/dl (up from 1774 mg/dl), and 2757 mg/dl (up from 1606 mg/dl). Individual levels and courses are also shown in Fig. 1.

### Disease activity

Muscle enzymes remained stable and within normal limits. Patient 1 experienced mild worsening of the CMAS (from 51 to 49), which improved again when switched to fSCIg five days apart. Patient 2 had mild, stable residual disease despite switching to fSCIg five days apart. Patients 3 and 4 maintained clinically inactive disease and kept fSCIg 14 days apart. Patient 5 had resolution of skin disease but mild residual muscle weakness with fSCIg every 14 days which resolved after switching to fSCIg five days apart.

### Subcutaneous immune globulin administration and monitoring for adverse effects

During and after fSCIg administration, the patients developed a localized subcutaneous pocket which resolved within 24 h. One patient (Patient 5) had transient mild headaches following administration of SCIg and a minor local site reaction. None of the patients required premedications (which all had required while receiving IVIg). There were no incidences of treatment abortion due to adverse effects. In three patients fSCIg five or 14 days apart was tolerated equally well. There was no evidence of dystrophic calcification at the site of fSCIg administration or elsewhere. All five patients reported that they strongly preferred treatment with fSCIg over IVIg, mostly due to the lack of adverse effects and the avoidance of hospitalization for i.v. therapy.

## Conclusions

This is, to our knowledge, the first report on the treatment of severe refractory JDM with high-dose fSCIg. The application of standard preparations of SCIg (without recombinant human hyaluronidase) in moderately severe JDM (up to 1 g/kg and month) and refractory adult DM (up to 0.8 g/kg and month) resulting in improved quality of life has been reported previously [14–16]. While standard SCIg is typically applied between one and three times weekly (in clinical practice maximally 56 g per month), fSCIg may be applied less frequently, e.g., once monthly in case of primary immunodeficiency, since it shows a better bioavailability and in addition allows administration of up to 120 g of Ig per month [13]. We demonstrated that the administration of two doses of fSCIg separated by five days for a total dose of 2 g/kg and month results in peak serum IgG levels similar to those achieved by regular high-dose IVIg treatment but not by conventional SCIg treatment. It is suggested that high peak serum IgG levels may be necessary for immune modulatory efficacy of Ig therapy [10, 11]. We were able to avoid premedication before IgG application and used oral corticosteroids sparingly to avoid long-term steroid adverse effects.

High-dose fSCIg therapy was well tolerated by our patients and even when given five days apart producing high IgG serum levels, we did not observe any relevant local or systemic adverse effects. Specifically, there was no evidence of increased local inflammation or dystrophic calcification at the administration sites and no evidence of aseptic meningitis or severe headaches. While high-dose IVIg treatment typically requires hospitalization and intravenous access, fSCIg can be administered without difficulty at home and may therefore be both time- and cost-saving [13, 17].

Our report is limited by the fact that this is a retrospective analysis of a small cohort of patients so that the assessment of treatment efficacy is very limited.

In summary, high-dose fSCIg therapy may be an attractive treatment option for patients with moderately severe or severe refractory JDM as a remission maintenance treatment and potentially also as a remission induction therapy in case of IVIg intolerance, IgA deficiency or difficult venous access, in order to improve quality of life (avoiding hospital admissions) and to reduce cost of treatment. Generally, the use of IVIg or fSCIg in JDM may allow reduction of steroid use. Further formal testing of efficacy and safety by means of prospective clinical trials is desirable.

## Abbreviations

CMAS: Childhood myositis assessment scale
fSCIg: Hyaluronidase-facilitated subcutaneous immune globulins
Ig: Immune globulin
IVIg: Intravenous immune globulins
JDM: Juvenile dermatomyositis
SCIg: Subcutaneous immune globulins
